# Study of Cationic Surfactants Raw Materials for COVID-19 Disinfecting Formulations by Potentiometric Surfactant Sensor

**DOI:** 10.3390/s23042126

**Published:** 2023-02-13

**Authors:** Nikola Sakač, Dubravka Madunić-Čačić, Dean Marković, Marija Jozanović

**Affiliations:** 1 Faculty of Geotechnical Engineering, University of Zagreb, 42000 Varaždin, Croatia; 2 Saponia Chemical, Pharmaceutical and Foodstuff Industry, Inc., 31000 Osijek, Croatia; 3 Department of Biotechnology, University of Rijeka, 51000 Rijeka, Croatia; 4 Department of Chemistry, University of Osijek, 31000 Osijek, Croatia

**Keywords:** quaternary ammonium compounds, QAC, COVID-19, cationic surfactant, disinfectant, potentiometric sensor, SARS-CoV-2

## Abstract

The behavior of a new 1,3-dioctadecyl-1*H*-imidazol-3-ium tetraphenylborate (DODI-TPB) surfactant sensor was studied in single and complex mixtures of technical grade QACs—benzalkonium chloride (BAC), *N,N*-didecyl-*N,N*-dimethylammonium chloride (DDAC), and *N,N*-dioctyl-*N,N*-dimethylammonium chloride (DOAC) usually used in COVID-19 disinfecting agents formulations. The results obtained with the new DODI-TPB sensor were in good agreement with data measured by a 1,3-dihexadecyl−1*H*-benzo[*d*]imidazol−3-ium-tetraphenylborate (DMI-TPB) surfactant sensor, as well as two-phase titration used as a reference method. The quantitative titrations of a two-component mixture of the cationic homologs (a) DDAC and DOAC; and (b) BAC and DOAC showed that the new DODI-TPB surfactant sensor can clearly distinguish two separate mixture components in a single potentiometric titration curve with two characteristic inflexion points. The consumption of SDS (used as a titrant) in the end-point 1 (EP 1) corresponded to the content of DDAC (or BAC), whereas the consumption in the end-point 2 (EP 2) corresponded to the total content of both cationic surfactants in the mixture. DOAC content in both mixtures can be calculated from the difference of the titrant used to achieve EP1 and EP2. The addition of nonionic surfactants resulted in the signal change decrease from 333.2 mV (1:0; no nonionic surfactant added) to 243.0 mV (1:10, *w*/*w*). The sensor was successfully tested in ten two-component COVID-19 disinfecting formulations.

## 1. Introduction

Surfactants are key ingredients used as emulsifiers, dispersing, cleaning, wetting, foaming, and anti-foaming agents in many household, and industrial applications and products. Surfactants are most often amphiphilic organic compounds containing a hydrophilic head and a hydrophobic tail. Whereas the nonionic surfactants are characterized by the absence of the charged groups in their head, the ionic surfactants can be divided into cationic, anionic, and amphoteric. Cationic surfactants are mainly used as disinfectants and antiseptics in households as well as industrial cleaning agents. During the COVID-19 pandemic, most disinfectants or antiseptics used for hand and surface disinfection were based on alcohol or cationic surfactants or on various combinations (alcohol-quaternary formulations) [1]. SARS-CoV-2 and other corona-type viruses have an envelope with a protective lipid layer derived from the host, which ensures the survival and transmission of the virus [2,3,4]. Alcohol and cationic surfactant-based disinfectants target the envelope to inactivate and disintegrate the virus [5]. Alcohol is usually used in high concentrations; it is flammable, dries the skin, and has a short activity lifetime on the surface since it evaporates quickly [6]. Even though alcohols are effective in annihilating infectious microorganisms, they are not sporicidal. Other disinfectants, such as quaternary ammonium compounds (QACs), glutaraldehyde, and hydrogen peroxide, outshine alcohol due to the listed benefits [7]. Therefore, alcohol-based disinfectants are generally not used to disinfect equipment and environments in healthcare facilities, but are often combined with other biocidal substances. Cationic surfactants are known to interact with DNA–RNA molecules, proteins, and lipids from viruses, resulting in virus disintegration [8]. Because of this specific interaction, they are also used in surfactant-based therapy against the COVID-19 disease [5,9,10]. For these reasons, the demand for cationic surfactants has rapidly increased [11]. Even though in the current COVID-19 pandemic the positive effects of cationic surfactants are more than welcome, it is important to emphasize the negative side effects of the cationic surfactants on the environment [1,12] and living organisms, especially humans [13]. Ignorance of the negative aspects of the cationic surfactants use and a lack of experience in handling disinfectants in the general population can lead to improper use of disinfectants and cause problems for the skin, the respiratory system, and the environment [13,14]. Cationic surfactants are also used in the food industry for the decontamination and prevention of infections spreading. For example, benzyldimethyldodecylammonium chloride, benzyldimethyltetradecylammonium chloride, and benzyldimethylhexadecyl ammonium chloride are used as surface decontaminants inside milk tanks. Therefore, the EU regulation of 0.01 mg/kg QAC residue should be monitored in food processing [15]. For all these reasons, it is important to raise public awareness and to establish reliable analytical tools [16,17,18] to quantify cationic surfactants during the production process in the final products and environment. The usual method for cationic surfactants quantification is the classical two-phase titration [19], in which a color change occurs when cationic surfactants are present. This method is slow, unreliable, and requires toxic solvents. Since the concentration of cationic surfactants in samples can be high, as in commercial products such as disinfectants, or low, as in wastewater or regular waters, there is a need to establish methods that can measure the amounts of cationic surfactants over a wide range of concentrations. Chemical sensors based on the PVC-based liquid membrane incorporated with an ionophore offer such an advantage [20]. In addition, they are inexpensive, respond within seconds, are easy to make and use, and do not require toxic solvents. The use of nanomaterials [21,22] and tuning of content and the plasticizer type [23] in the development of PVC-based surfactant sensors could have a positive impact on stability, longer lifetime, and slightly better detection limits. The ionophore plays a crucial role in the fabrication of sensing membranes. For this reason, it is important to synthesize new ionophores [24], which could lead to the preparation of surfactant sensors with high stability and sensitivity, as well as advanced selectivity.

Commercial product formulations for disinfectants and antiseptics use quaternary ammonium compounds, a type of cationic surfactant, as the active component. QACs are inexpensive agents and are commonly used as disinfectants and antiseptics for home and professional use [25,26]. QACs could be used in formulations with nonionic surfactants to enhance their disinfecting and cleaning properties.

QACs are an optimal choice for disinfection, preservation, cleaning, and antiseptic activity in hospitals and other facilities because they are cost-effective and act rapidly against a broad range of microorganisms. For example, QACs are active components in more than 200 disinfectants listed in the recently published EPA (Environmental Protection Agency) list [27]. Consequently, these compounds are an essential component of infection control practices and the prevention of nosocomial infections in medical facilities [28]. Some of the most widely used cationic surfactants found in disinfectants are benzalkonium chloride (BAC) [29], *N,N*-didecyl-*N,N*-dimethylammonium chloride (DDAC) [30], alkyl dimethyl benzyl ammonium saccharinate, and cetyl pyridinium chloride [31]. Because of the high potency of cetylpyridinium chloride and miramistin against a broad spectrum of viruses, Baker et al. suggested that clinically approved mouthwashes or nasal sprays could help to reduce the transmission of SARS-CoV-2 [32].

Furthermore, due to the high mutation rate of viruses and bacteria, and the constant emergence of new strains, there is a great need for new effective QACs. Therefore, a new generation of twin-chain or dialkyl quaternaries such as didecyldimethylammoniumbromide [33] and dioctyldimethylammonium bromide [34], which are virucidal even in hard water and in the presence of anionic residues, has been developed. The addition of alkyl or aromatic groups within the R group also alters the function of the QAC. Thus, QACs with methyl groups in positions from C12 to C16 exhibit the strongest antimicrobial activity. Several studies have demonstrated the potent activity of QACs against influenza and SARS viruses [35,36]. Karamov et al. investigated a series of cationic surfactants for their disinfecting activity against the SARS-CoV-2 virus [2]. They concluded that cationic surfactants with greater length and a number of hydrophobic tails, and with benzene in the structure could increase the virucidal effect on the SARS-CoV-2 virus. For example, cationic surfactants didodecyldimethylammonium bromide and benzalkonium chloride deactivate the SARS-CoV-2 virus in only 5 s.

QACs using disinfectants are able to effectively inactivate viruses even in the presence of organics, unlike other common disinfectants such as alcohol and chlorine-based disinfectants, whose effectiveness is reduced by the presence of organic matter [30]. Another advantage of cationic surfactants is the ability to combine them with a variety of cleaning agents to achieve both a cleaning and a disinfecting effect. Nyco’s Sani-Spritz spray disinfectant is an example of a QAC-based disinfectant that provides both cleaning properties and broad-spectrum antimicrobial activity for many common and dangerous bacteria and viruses (including emerging pathogens and SARS-CoV-2).

In our recent work, we presented and characterized a new 1,3-dioctadecyl-1*H*-imidazol-3-ium tetraphenylborate ion-pair prepared from the new QAC 1,3-dioctadecyl-1*H*-imidazol-3-ium cation [37]. This ion-pair was used to prepare a surfactant sensor based on a DODI-TPB [38] PVC liquid membrane. The aim of the current work is to study the behavior of the developed DODI-TPB surfactant sensor in complex mixtures of technical grade cationic surfactants used in commercial disinfectants; then, to test it for the interfering effect of nonionic surfactants normally found in commercial product formulations; and finally, to use it for the quantification of cationic surfactants in commercial COVID-19 disinfectants and antiseptics for home and professional use.

## 2. Materials and Methods

### 2.1. Reagents and Materials

Chemicals for the 1,3-dioctadecyl-1*H*-imidazol-3-ium bromide (DODI-Br) cationic ion synthesis were 1-bromooctadecane, with 1*H*-imidazole and NaHCO_3_ (Sigma-Aldrich, Hamburg, Germany). Chemicals for the 1,3-dioctadecyl-1*H*-imidazol-3-ium tetraphenylborate (DODI-TPB) ion-pair synthesis were a DODI-Br and a sodium tetraphenylborate (TPB) (Fluka, Buchs, Switzerland) used as a counter ion. All the chemicals were analytical grade.

Ionophore was prepared according to the procedure recently described [37]. A PVC-based liquid-type sensing membrane was prepared by mixing the high molecular weight PVC (Sigma-Aldrich, Hamburg, Germany); a selected hydrophobic plasticizer *o*-nitrophenyloctylether (*o*-NPOE) (Sigma-Aldrich, Hamburg, Germany) with tetrahydrofuran (THF) (Merck, Darmstadt, Germany), all analytical grade; and a DODI-TPB ion-pair.

Anionic surfactant dodecyl sulfate (DDS) (Fluka, Buchs, Switzerland), analytical grade, was used as a titrant for all the potentiometric titrations.

Technical grade cationic surfactants (with abbreviations) used as analytes for potentiometric titrations, with their corresponding commercial names, are shown in Table 1.

The nonionic surfactant used for the study of the influence of nonionic surfactants on titrations of cationic surfactant was analytical grade Triton X-100 (Merck, Darmstadt, Germany).

pH was adjusted by adding the corresponding amounts of NaOH and HCl (all from Kemika, Croatia).

Ultrapure water was used for all the dilutions, solution preparations, and measurements.

### 2.2. Measuring Setup

All the potentiometric titrations were carried out by a Metrohm 808 Titrando titrator with a stirrer and Metrohm Tiamo software (all from Metrohm, Herisau, Switzerland). The electrode-measuring system consisted of a silver/silver (I) chloride electrode with potassium chloride (3 M) electrolyte (Metrohm, Herisau, Switzerland) reference electrode and a DODI-TPB surfactant sensor made of a Philips electrode body IS-561 (Glasblaeserei Moeller, Zurich, Switzerland), 3 M NaCl inner solution, and a PVC-based liquid membrane with a DODI-TPB ionophore mounted on the bottom of the electrode body.

### 2.3. Potentiometric Titrations Procedure

Potentiometric titrations were employed to observe the behavior of the DODI-TPB (as an end-point indicator) in complex model samples of technical grade cationic surfactants and in model samples of technical grade cationic surfactants containing different amounts of interfering nonionic surfactants. The DODI-TPB surfactant sensor was also used as an end-point indicator to quantify the amount of cationic surfactants in commercial disinfectant samples. The measuring parameters during potentiometric titrations were fixed to the dynamic equivalence point titration mode (DET mode) with limited signal drift to 5 mV/min. Increments were added according to the dynamic behavior of the measuring signal and a corresponding calculated slope during the titration.

Titrations of technical grade cationic surfactants BAC, DDAC, and DOAC were performed by DODI-TPB as an end-point indicator and an anionic surfactant SDS (*c* = 4 mM) as a titrant.

Potentiometric titrations of the two-component model mixtures of DDAC and DOAC [1:1 (*w*/*w*)] and of BAC and DOAC (1:1 *w*/*w*) were performed by DODI-TPB as an end-point indicator and an SDS (*c* = 4 mM) as a titrant.

Potentiometric titrations of model mixtures of DDAC and non-ionic surfactant Triton X-100 in different proportions 1:0, 1:1,1:2, 1:3, 1:5, 1:10 (*w*/*w*) were performed using SDS (*c* = 4 mM) as a titrant and a DODI-TPB potentiometric sensor as an end-point indicator.

Potentiometric titrations of the two-component COVID-19 formulations were performed by DODI-TPB as an end-point indicator and an SDS (*c* = 4 mM) as a titrant.

After each measurement, electrodes were rinsed in deionized water and dried. The measurements were performed at room temperature.

## 3. Results

In this investigation, the surfactant sensor DODI-TPB was used as an indicator and endpoint detector in potentiometric titrations of several selected technical cationic surfactants commonly used in detergents and disinfectants for public facilities, hospitals, and industry; then, in titrations of two-component model mixtures; and finally, to determine the cationic surfactants in several COVID-19 formulations of disinfectants. In all the titrations, SDS (*c* = 4 mM) was used as the titrant.

### 3.1. Titrations of Technical-Grade Cationic Surfactants

The DODI-TPB sensor for surfactants showed an excellent response to cationic surfactants with a nearly Nernstian response and a wide linear concentration range (up to 1.8 × 10^−6^ to 1.0 × 10^−4^ M) [37]. The titration curves of the pure cationic surfactants showed a sigmoidal shape, a high signal potential change, and well-defined inflexion points [37]. Therefore, the surfactant sensor DODI-TPB was tested on samples of technical grade cationic surfactants used as raw materials in the production of disinfectants and antiseptics.

**Figure 1:**
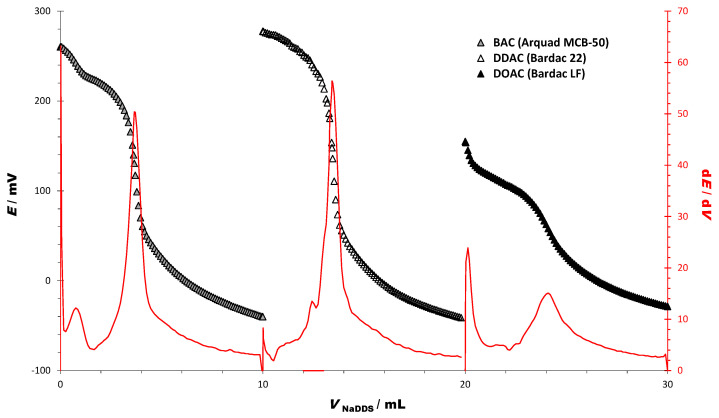
Potentiometric titration curves and their first derivatives of three cationic surfactants: BAC, DDAC, and DOAC using SDS as a titrant (*c* = 4 mM) and DODI-TPB potentiometric sensor as an indicator.

The DODI-TPB sensor was used as an indicator and endpoint detector in titrations of the technical-grade BAC, DDAC, and DOAC (Table 1), employing SDS (*c* = 4 mM) as a titrant, and showed excellent response properties (Figure 1).

Three cationic surfactants were studied and all exhibited sigmoidal curves with a sharp signal change and well-defined inflexion at the equivalence points (Table 2). The titration curve for BAC is well defined, with a signal change up to 300.9 mV. Comparing DDAC and DOAC titration curves, it can be observed that these two very similar cationic surfactants (differing only in the length of the fatty alkyl chains) obtained different titration curves. The titration curve for DDAC is very sharp, with an exceptional signal change of up to 319.78 mV. The titration curve for DOAC is sigmoidal and has a well-defined inflexion point; however, the signal change is much smaller compared to the DDAC titration curve up to 183.61 mV. This is an indication that the sensor is able to detect the signal change corresponding to the type of cationic surfactant used. Corresponding first derivatives (dE/dV) for all the titration curves showed a high signal change at the equivalence point with a sharp peak, ensuring high accuracy and precision in the determination (Table 2).

The results obtained with the sensor DODI-TPB were compared with a previously developed and well-designed sensor for cationic surfactants DMI-TPB [39] and a reference two-phase titration method, used as a standard method for the quantification of cationic surfactants [40]. The content of cationic surfactant determined by the DODI-TPB sensor was 51.70% for BAC, 50.06% for DDAC, and 50.52% for DOAC. The RSDs for all three cationic surfactants were less than 0.5%. The results showed good agreement with the new surfactant sensor DODI-TPB, sensor DMI-TPB, and the reference method—two-phase titration.

The results for the potentiometric determination of selected technical cationic surfactants by SDS (*c* = 4 mM) used as titrant and the DODI–TPB sensor for ionic surfactants as an indicator, in comparison with the results obtained with the DMI-TPB ionic-surfactant sensor and the standard method of two-phase titration and the corresponding statistics, are shown in Table 3.

### 3.2. Titrations of Cationic Surfactants in Two-Component Model Mixtures

**Figure 2:**
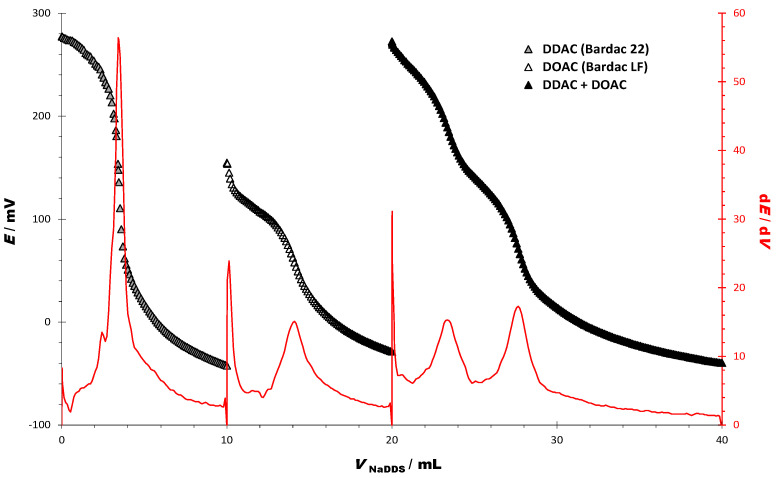
Potentiometric titration curves and their first derivatives of DDAC, DOAC, and the two-component model mixture of DDAC and DOAC [1:1 (*w*/*w*)], using SDS as titrant (*c* = 4 mM) and DODI-TPB potentiometric sensor as an indicator.

From the titration curves of the pure solutions of BAC, DDAC, and DOAC, it is evident that there is a significant difference between the cationic properties of the DOAC and DDAC (Figure 1, Table 2). We exploited these properties to observe the response of the sensor DODI-TPB when these two cationic surfactants are present in the same mixture. Potentiometric titration of the two-component mixture of the cationic homologues DDAC and DOAC yields a differential titration curve with two distinct inflections (Figure 2).

The consumption of the titrant in EP 1 (mL) corresponds to the content of DDAC, while the consumption of the titrant in EP 2 (mL) corresponds to the content of both cationic surfactants in the mixture. The DOAC content corresponds to the difference in consumption between EP1 and EP2. This is significant since the response of the surfactant sensor DODI-TPB can clearly distinguish two separate mixture components in a single potentiometric titration curve. The quantitative results for the titration of the two-component mixture of the cationic homologs DDAC and DOAC are presented in Table 4.

Although the potential change in the titration of the two-component mixture is the same as in the titration of DDAC (about 272 mV), the endpoint values for the individual cationic surfactants (DDAC and DOAC) correspond to the endpoint values in the two-component mixture, with well-defined inflexions (Figure 2).

**Figure 3:**
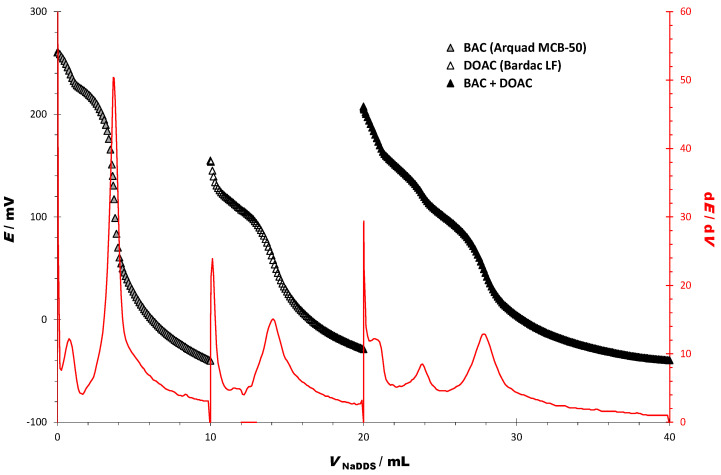
Potentiometric titration curves and their first derivatives of BAC, DOAC, and the two-component model mixtures of BAC and DOAC (1:1 *w*/*w*), using DDS as titrant (*c* = 4 × 10^−3^ mol/L) and DODI-TPB potentiometric sensor as an indicator.

Titration of BAC and DOAC was performed in the same way. As expected, the titration was very similar, even with two inflections (Figure 3). Consumption of the titrant in EP 1 (mL) corresponds to the content of BAC, while consumption of the titrant in EP 2 (mL) corresponds to the content of both cationic surfactants in the mixture. The DOAC content corresponds to the consumption difference between EP1 and EP2 (mL).

The potentiometric properties of the titration curves for model solutions of BAC, DOAC, and a separate two-component mixture of BAC and DOAC (1:1 *w*/*w*) are presented in Table 5. The values shown are the starting and an end potential (mV), and ΔE (mV) for titration curves and corresponding EP values for the first derivative.

The results for the potentiometric quantification of cationic surfactants in two-component model mixtures containing DDAC and DOAC (shown in Figure 2), and BAC and DOAC (shown in Figure 3), are shown in Table 6. The calculated values for the two-component model mixtures ranged from 48.18 to 53.43%, with a corresponding recovery of 96.24 to 105.76%, respectively.

### 3.3. Influence of Non-Ionic Surfactants on the Titration of Cationic Surfactants

**Figure 4:**
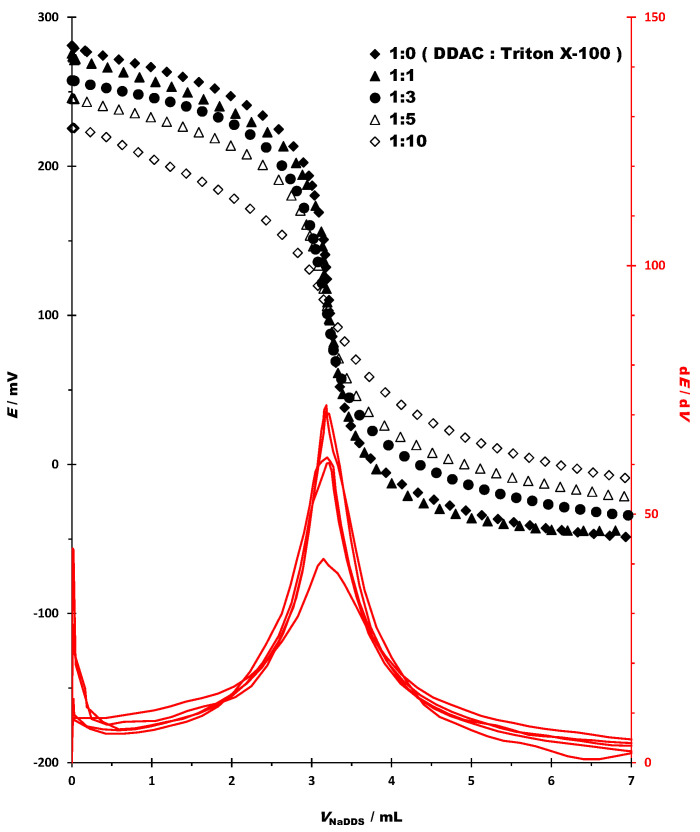
Potentiometric titration curves and their first derivatives of the model mixtures of DDAC and nonionic surfactant Triton X-100 in different proportions (*w*/*w*), using NaDDS as titrant (*c* = 4 × 10^−3^ mol/L) and DODI-TPB as a potentiometric sensor.

Sometimes nonionic surfactants are included in the formulations of disinfectants and antiseptics with cationic surfactants. The influence of the nonionic surfactant Triton X-100 in different proportions (*w*/*w*) was observed in the potentiometric titration with DDAC by anionic surfactant NaDDS (*c* = 4 × 10^−3^ M) and the potentiometric sensor DODI-TPB as an indicator (Figure 4).

Increased amounts of nonionic surfactant Triton X-100 decreased the bending of the titration curve, reduced the signal change, and decreased the value of the first derivative at the endpoint. The quantitative characteristics of the titration curve for the potentiometric titrations of the model mixtures of DDAC and the nonionic surfactant Triton X-100 in different proportions (*w*/*w*), using NaDDS as the titrant (*c* = 4 × 10^−3^ M) and the potentiometric sensor DODI-TPB as an indicator, are presented in Table 7.

The addition of nonionic surfactants resulted in a decrease of the signal change from 333.2 mV (1:0; no nonionic surfactant added) to 243.0 mV (1:10, *w*/*w*). The change in the first derivative signal corresponded to the change in the potential signal. The decrease in the change of the first derivative ranged from 71.9 (1:0; with no nonionic surfactant added) to 43.0 dE/dV (1:10, *w*/*w*). Although the nonionic surfactants have a negative effect on the response of the surfactant sensor to DDAC, the usual concentration range for cationic surfactants in commercial products is up to 1:2 (cationic surfactant: nonionic surfactant, *w*/*w*). In this way, the nonionic surfactants could have a negative impact, but still with well-defined endpoints.

### 3.4. Potentiometric Determinations of Cationic Surfactants in Commercial Samples

Ten samples of COVID-19 disinfectant formulations with DDAC and DOAC (six samples), and BAC and DOAC (four samples) were titrated using NaDDS as a titrant (*c* = 4 × 10^−3^ M) and the DODI-TPB potentiometric sensor as the endpoint indicator. The titration results with the corresponding contents of each cationic surfactant are shown in Table 8. The first EP1s corresponded to the content of DDAC and BAC, while the second EP2s corresponded to the sum of DOAC and certain cationic surfactants in the two-component mixture. The sum of the cationic surfactant contents (%) for the first six samples ranged from 4.350 to 5.596%, while the sum for the other four samples with BAC ranged from 2.361 to 3.665. The recoveries calculated from the values for the reference two-phase filtration method and the sensor DODI-TPB ranged from 96.4 to 102.2%.

## 4. Conclusions

The potentiometric surfactant sensor based on the new ion-pair DODI-TPB has been successfully employed for the quantification of technical-grade cationic surfactant BAC, DDAC, and DOAC, which are used as raw materials for COVID-19 disinfectant formulations. Potentiometric titration results for two-component mixtures of the cationic homologs a) DDAC and DOAC; and b) BAC and DOAC showed that the DODI-TPB surfactant sensor can clearly distinguish two individual mixture components in a single potentiometric titration curve. In potentiometric titrations of DDAC, increased amounts of nonionic surfactant Triton X-100 had a negative effect on the inflexion of the titration curve, decreased the signal change, and lowered the value of the first derivative at the endpoint. The quantitative results of the BAC, DDAC, and DOAC in COVID-19 disinfectant formulations showed good agreement with the standard two-phase titration method. The presented study is an interesting tool not only for quantification of the cationic surfactants in raw materials, but also for discrimination and quantification of similar cationic surfactants in their mixtures.

## Figures and Tables

**Table 1:** The chemical names, abbreviations, commercial names, and declared mean M_r_ of three technical grade cationic surfactants investigated.

SURFACTANT INVESTIGATED				
Chemical Name	Abbreviation	Commercial Name	Mean M_r_ Declared	Manufacturer/ Country
Benzalkonium chloride	BAC	Arquad MCB-50	352.5	AkzoNobel/ Netherlands
*N,N*-Didecyl-*N,N*-dimethylammonium chloride	DDAC	Bardac 22	361.0	Lonza/ Swisserland
*N,N*-Dioctyl-*N,N*-dimethylammonium chloride	DOAC	Bardac LF	312.0	

**Table 2:** Characteristics of the titration curves of the technical grade cationic surfactants: BAC, DDAD, and DOAC.

CHARACTERISTICS OF THE TITRATION CURVES	BAC	DDAC	DOAC
Starting potential (E/mV)	260.16	277.44	154.84
Ending potential (E/mV)	−40.2	−42.34	−28.77
ΔE/mV	−300.9	−319.78	−183.61
**End point**			
dE/dV (End point)	50.4	56.4	15.1
EP (E/mV)	130.52	147.94	58.00

**Table 3:** Results of potentiometric determination of some technical cationic surfactants using SDS (*c* = 4 mM) as a titrant and DODI-TPB as an indicator for ionic surfactants compared to results obtained with the DMI-TPB sensor for ionic surfactants and the standard two-phase titration method.

Surfactant Used	SURFACTANT CONTENT *							
	DODI-TPB Sensor			DMI-TPB Sensor [39]			Two-Phase Titration [40]	
	Found (%)	RSD (%)	Rel. Error (%)	Found (%)	RSD (%)	Rel. Error (%)	Found (%)	RSD (%)
BAC	51.70	0.226	1.63	51.88	0.195	1.99	50.87	0.563
DDAC	50.06	0.174	−0.64	51.77	0.207	2.76	50.38	0.608
DOAC	50.52	0.433	−1.81	50.88	0.460	−1.11	51.45	1.151

* Average of three determinations.

**Table 4:** Characteristics of the titration curves of model solutions of DDAC, DOAC, and the two-component mixture of BAC and DOAC (1:1 *w*/*w*).

CHARACTERISTICS OF THE TITRATION CURVES	DDAC	DOAC	DDAC + DOAC
Starting potential (E/mV)	277.44	154.84	272.67
Ending potential (E/mV)	−42.34	−28.77	−39.73
ΔE/mV	−319.78	−183.61	−312.40
**End point (EP)**			
dE/dV (EP1)	56.4	15.1	15.3
dE/dV (EP2)	-	-	17.3
EP 1 (E/mV)	147.94	58.00	189.04
EP 2 (E/mV)	-	-	71.18

**Table 5:** Characteristics of titration curves of model solutions of BAC, DOAC, and the two-component model mixtures of BAC and DOAC (1:1 *w*/*w*).

CHARACTERISTICS OF THE TITRATION CURVES	BAC	DOAC	BAC + DOAC
Starting potential (E/mV)	260.16	154.84	247.22
Ending potential (E/mV)	−40.2	−28.77	−39.67
ΔE/mV	−300.9	−183.61	−247.22
**End point (EP)**			
dE/dV (EP1)	50.4	15.1	8.5
dE/dV (EP2)	-	-	12.9
EP 1 (E/mV)	130.52	58.00	121.65
EP 2 (E/mV)	-	-	46.08

**Table 6:** Results of potentiometric determination of the content of cationic surfactants in two-component model mixtures containing DDAC + DOAC, and BAC and DOAC.

			Expected (%)	Obtained (%) *	Recovery (%)
	Two-Component Mixture DDAC + DOAC				
DDAC			50.06	48.18	96.24
DOAC			50.52	53.43	105.76
	**Two-Component Mixture BAC + DOAC**				
BAC			51.70	53.69	103.86
DOAC			50.52	50.42	99.81

* Average of three determinations.

**Table 7:** Characteristics of the titration curves of the model mixtures of DDAC and nonionic surfactant Triton X-100 in different proportions (*w*/*w*), using NaDDS as titrant (*c* = 4 × 10^−3^ M) and DODI-TPB as a potentiometric sensor.

CHARACTERISTICS OF THE TITRATION CURVES	DDAC: Triton X-100 (m/m)				
	1:0	1:1	1:3	1:5	1:10
ΔE/mV	333.2	320.3	298.5	274.4	243.0
dE/dV	71.9	71.0	61.4	60.3	41.0

**Table 8:** Potentiometric determinations of cationic surfactants in COVID-19 disinfecting formulations using NaDDS as a titrant (*c* = 4 × 10^−3^ M) and the potentiometric sensor DODI-TPB as an endpoint indicator.

	DODI-TPB Sensor (%)			Two-Phase Titration (%) *	Recovery (%)
	DDAC (EP1)	DOAC (∆EP)	Sum (EP2)		
Sample 1	2.031	3.413	5.444	5.325	102.2
Sample 2	1.526	3.521	5.047	5.073	99.4
Sample 3	3.672	1.245	4.917	5.061	97.1
Sample 4	2.677	1.673	4.350	4.275	101.7
Sample 5	0.974	4.622	5.596	5.612	99.7
Sample 6	2.856	1.956	4.812	4.728	101.7
	**BAC (EP1)**	**DOAC (∆EP)**	**Sum (EP2)**		
Sample 7	2.367	1.264	3.631	3.602	100.8
Sample 8	1.362	1.374	2.736	2.838	96.4
Sample 9	0.934	1.427	2.361	2.381	99.1
Sample 10	0.882	2.783	3.665	3.751	97.7

* Average of three determinations.

## Data Availability

Not applicable.
